# Beyond Moderate to Vigorous Physical Activity (MVPA): Why Physical Activity Surveillance May Be Failing Culturally and Linguistically Diverse (CALD) Girls in Australia

**DOI:** 10.1002/hpja.70223

**Published:** 2026-07-27

**Authors:** Kaushik Talukdar

**Affiliations:** ^1^ School of Health Sciences Western Sydney University Sydney Australia

**Keywords:** accelerometry, adolescent health, culturally and linguistically diverse (CALD), health equity, movement behaviours, physical activity surveillance

## Abstract

Physical inactivity among culturally and linguistically diverse (CALD) girls in Australia is typically framed as a participation problem. However, emerging evidence suggests it is equally a measurement problem. Current surveillance systems rely heavily on self‐report tools, sport‐centric participation models and generic moderate‐to‐vigorous physical activity (MVPA) thresholds that may underestimate meaningful movement exposure whilst failing to detect genuine developmental risk. Longitudinal data show that CALD girls accumulate the lowest MVPA across adolescence, yet these patterns may reflect both true inactivity and under‐recognition of culturally grounded movement practices. Self‐report instruments often overlook transport‐related activity, household responsibilities, caregiving‐related movement and traditional dance forms involving repeated loading, muscular endurance, balance and multidirectional movement. These activities carry strength‐related and musculoskeletal relevance but remain largely invisible within mainstream surveillance frameworks. Accelerometry improves objectivity but is still interpreted through MVPA‐centric cut‐points that do not capture force production, impact loading or mechanical competence—key components of adolescent development and long‐term participation capacity. Reframing physical inactivity among CALD women as a downstream consequence of early‐life exclusion from movement systems shifts the focus from participation to prevention. Improved measurement—through raw accelerometry, machine‐learning–based activity recognition and ecological momentary assessment—offers a pathway to more accurate surveillance and more equitable policy responses. CALD girls are not only underrepresented in physical activity systems; they are often under‐measured within them. Addressing surveillance blind spots is essential for designing prevention‐focused, culturally responsive, developmentally meaningful interventions.

## 1 Introduction

Physical inactivity remains one of Australia's most persistent and costly public health challenges. Recent modelling suggests insufficient physical activity costs the Australian economy up to AUD $15.6 billion annually through productivity losses, health care expenditure and premature mortality [1]. Updated burden of disease analyses indicate that AUD $2.4 billion of health system expenditure in 2018–2019 was attributable to diseases linked to insufficient physical activity, with coronary heart disease, falls and depressive disorders accounting for the largest proportions [1]. Physical inactivity contributes approximately 2.1% of Australia's total burden of disease, reinforcing its significance as a population‐level public health issue [1].

Whilst physical inactivity is often framed as a participation problem, emerging evidence suggests that for some populations it may also represent a surveillance problem. This is particularly relevant for culturally and linguistically diverse (CALD) girls, who appear to experience both lower participation in mainstream physical activity systems and potential under‐recognition within the surveillance tools used to measure activity. Understanding this dual challenge is essential for designing equitable policy and intervention responses.

These economic and health consequences sit alongside a concerning national participation profile. The Australian Institute of Health and Welfare (AIHW) reports that 83% of adolescents aged 15–17 years do not meet physical activity guidelines, whilst 80% fail to achieve recommended muscle‐strengthening activity levels [2]. Australian Bureau of Statistics (ABS) data further demonstrate a pronounced gender gap, with only 3.7% of girls aged 15–17 years meeting physical activity guidelines compared with 9.9% of boys. These findings are often presented as a broad adolescent inactivity problem [2, 3]. However, emerging evidence suggests the issue is more nuanced—and more unequal—than national averages imply.

A recent longitudinal analysis from the Longitudinal Study of Australian Children (LSAC) provides some of the strongest evidence to date. Adolescents from CALD backgrounds, particularly CALD girls, demonstrated the lowest levels of moderate‐to‐vigorous physical activity (MVPA) across all measurement waves, with declines accelerating across early and mid‐adolescence [4]. CALD girls accumulated an average of 73.4 min of MVPA at ages 10–11, declining to 37.3 min by ages 14–15—the lowest among all subgroups examined [4].

This pattern is often interpreted as evidence that CALD girls are simply ‘less active’. However, that conclusion may be incomplete. It assumes that our current surveillance systems accurately capture how CALD girls move, load and participate in physical activity. That assumption deserves greater scrutiny.

This commentary argues that physical activity inequity among CALD girls is not only a participation problem, but also a measurement problem. Current surveillance systems rely heavily on self‐report tools, sport‐centric participation models and generic MVPA thresholds that may systematically underestimate meaningful movement exposure whilst simultaneously failing to detect genuine risk [2, 3]. This argument does not diminish the importance of MVPA for cardiometabolic health; rather, it recognises that MVPA alone provides an incomplete picture of adolescent movement behaviour, particularly when strength development, mechanical loading, physical literacy and long‐term participation capacity are central to lifelong health outcomes. If we measure movement poorly, we risk designing ineffective interventions. In public health systems, what is measured shapes what is funded and what is funded shapes who remains visible in policy. We cannot solve inequity if we are measuring it inaccurately.

### 1.1 The Problem With How We Currently Measure Physical Activity

Most population surveillance of adolescent physical activity relies on self‐report questionnaires, recall‐based participation surveys or broad MVPA thresholds derived from accelerometry [2, 3]. Whilst useful for national surveillance, these approaches have important limitations, particularly when applied to culturally diverse populations.

Self‐report measures are vulnerable to recall bias, social desirability bias and cultural differences in how physical activity is interpreted [5]. Many instruments remain heavily oriented towards organised sport, leisure‐time exercise and Western recreational activity patterns. For CALD girls, whose movement experiences may be shaped by different cultural norms, family expectations, transport patterns, caregiving roles or community practices, these tools may fail to capture substantial components of daily movement exposure.

For example, walking for transport, physically demanding household responsibilities, caregiving‐related movement, community participation and culturally embedded movement practices such as traditional dance may not be consistently recognised or reported as ‘exercise’, despite meaningful physiological and musculoskeletal loading relevance. This creates a serious surveillance problem: low reported participation may reflect true inactivity, under‐recognition of meaningful movement, or both.

This distinction matters. If policy responses are built on incomplete measurement, interventions risk targeting symptoms rather than systems. Furthermore, although the available evidence suggests current approaches may inadequately capture important dimensions of movement among CALD girls, empirical validation studies are needed to quantify the extent of potential underestimation and determine which surveillance methods provide the most culturally valid assessment across diverse population groups.

### 1.2 MVPA Is Necessary, but Insufficient

The dominant public health lens remains centred on minutes of MVPA. Whilst this remains important for cardiometabolic health, it offers a narrow understanding of movement behaviour, particularly during adolescence.

For girls, and particularly for CALD girls navigating adolescence, physical activity is not only about energy expenditure. It is also about movement competence, confidence, musculoskeletal development and the development of strength‐oriented physical literacy. Activities involving jumping, landing, multidirectional movement, impact loading, resistance work and repeated force production may play an important role in bone health, injury prevention and long‐term participation capacity, even when they are not captured well through conventional MVPA frameworks.

This is particularly relevant when considering the transition from adolescence into adulthood. Girls who disengage from movement systems may lose not only aerobic activity exposure, but also opportunities to develop strength, confidence and mechanical competence. This has implications beyond sport participation, influencing long‐term engagement with exercise across the lifespan. Current surveillance frameworks rarely assess these movement characteristics well [6, 7].

### 1.3 Mechanical Loading Exposure Is Largely Invisible

One of the most overlooked limitations in physical activity surveillance is the poor measurement of mechanical loading exposure. Accelerometry can estimate movement intensity, but standard public health interpretation still prioritises time spent in MVPA rather than force production, impact loading or strength‐related exposure. This creates important surveillance blind spots.

Traditional dance forms common across many CALD communities—including South Asian classical dance traditions such as Bharatanatyam—often involve repeated lower‐limb loading, multidirectional movement, sustained muscular endurance, deep knee flexion, balance tasks and high‐volume rhythmic footwork [8, 9, 10]. These activities may provide meaningful strength‐related and musculoskeletal‐loading stimuli whilst also offering culturally familiar, socially acceptable and identity‐affirming movement opportunities.

Yet these forms of movement are rarely recognised within mainstream physical activity surveillance or policy language. They are often treated as cultural activities rather than legitimate strength‐building physical activity pathways. This is both a measurement failure and a policy failure.

For CALD girls who may not initially identify with Western sport pathways or gym‐based exercise models, culturally grounded movement practices may represent one of the most accessible entry points into lifelong physical activity. Ignoring these exposure risks both underestimates participation and misses opportunities for intervention.

**FIGURE 1:**
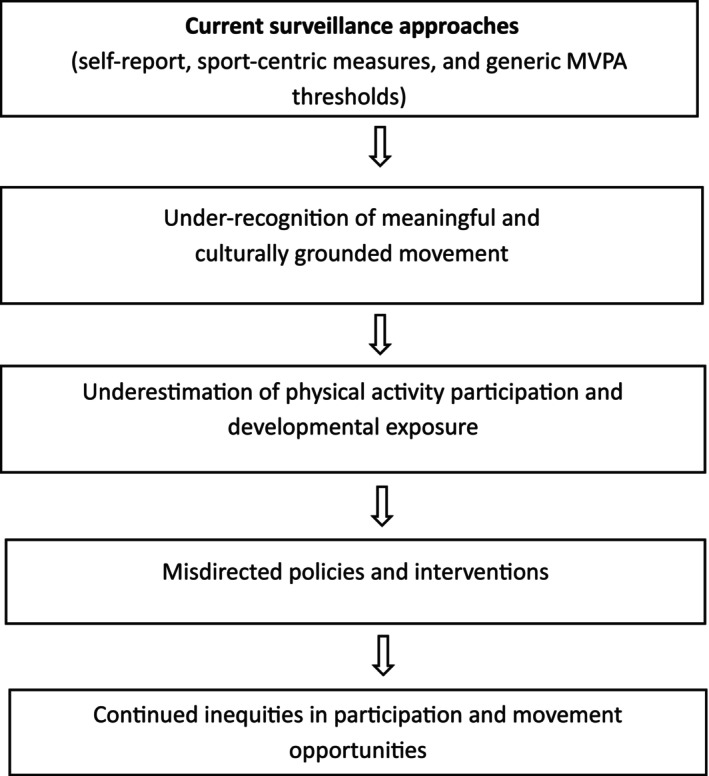
Proposed surveillance‐participation feedback loop illustrating how under‐recognition of meaningful movement may contribute to the persistence of participation inequities among CALD girls.

Future surveillance must move beyond traditional self‐report and generic accelerometer cut‐points towards methods capable of capturing both context and mechanical exposure. Raw accelerometry combined with machine learning‐based activity recognition offers one promising approach. Rather than reducing movement into broad intensity categories alone, these methods analyse continuous movement signals and can be trained to recognise specific activity patterns such as walking, running, jumping, dance‐based movement, stair climbing, resistance exercise or multidirectional movement. This provides richer information on how movement accumulates throughout the day and may improve recognition of culturally meaningful movement behaviours that conventional MVPA cut‐points often overlook [6, 7]. Hybrid models integrating accelerometry with ecological momentary assessment (EMA) or brief contextual reporting may further improve interpretation by identifying where movement occurs, how it is experienced, and whether it reflects culturally meaningful participation rather than isolated activity bouts [11]. Without more precise measurement, surveillance systems will continue to privilege what is easiest to count rather than what is most important for long‐term health and participation. The potential consequences of these surveillance limitations are illustrated in Figure 1, which outlines a proposed feedback loop whereby under‐recognition of meaningful movement may contribute to the underestimation of participation, misdirected intervention efforts and the persistence of inequities among CALD girls.

### 1.4 Why Cultural Adaptation Alone Is Not Enough

Australian policy has increasingly recognised diversity in sport and physical activity participation. National strategies addressing women and girls in sport and CALD participation emphasise inclusion, access and representation [12, 13]. However, these frameworks remain broad and often lack operational specificity for adolescent CALD girls.

Similarly, intervention research has shown that cultural adaptation alone is not enough.

A recent systematic review of culturally adapted physical activity interventions for CALD children and adolescents found that only a small number of interventions demonstrated significant improvements in physical activity outcomes, even when both surface‐level adaptations (e.g., translated resources) and deep cultural adaptations (e.g., integration of cultural values and practices) were used [14]. Across 20 studies, only 3 of 10 combined‐adaptation interventions, 1 of 6 surface‐only interventions and 1 of 4 deep‐only interventions demonstrated significant improvements in physical activity [14]. This is an important finding. It suggests that cultural adaptation alone may not be sufficient to improve physical activity outcomes among CALD children and adolescents. The review highlighted considerable heterogeneity in intervention design, delivery and outcome assessment, making it difficult to identify which adaptation strategies are most effective. It also identified a need for higher‐quality research to determine the optimal format, duration, frequency and implementation approaches for culturally adapted interventions.

These findings suggest that cultural tailoring should be viewed as an important component of intervention design rather than a standalone solution for addressing physical inactivity among CALD populations. More broadly, they highlight the need for future interventions to move beyond adaptation alone and examine how culturally responsive approaches can be integrated within wider participation systems and community contexts:

- Inclusion is not intervention.
- Representation is not retention.

### 1.5 From Participation to Prevention

Evidence suggests that physical inactivity among CALD women should not be framed primarily as an adult behavioural issue. It is more accurately understood as the downstream consequence of early‐life exclusion from movement systems during adolescence [4, 5].

CALD girls represent a critical prevention window where gendered, cultural, structural and measurement inequities converge [5, 14]. By the time adult interventions target participation, many of the foundational opportunities for confidence, competence and movement identity have already been lost [5, 14]. This reframing shifts the conversation from participation to prevention.

Schools remain the strongest universal prevention platform because they provide access before dropout occurs, but prevention cannot be outsourced to physical education alone [12, 13]. Sustainable engagement depends on coordinated pathways between schools, community sport systems, local councils and national participation strategies. Surveillance bodies, education systems and sporting organisations must align their definitions of participation and progression so that girls are not lost during the transition from compulsory school movement to voluntary community participation [6, 7]. Without this continuity, schools may identify the problem but cannot solve the retention failure alone.

School‐based systems can embed culturally responsive movement opportunities, strength‐based physical literacy and alternative pathways beyond traditional organised sport. Importantly, prevention must also extend beyond schools.

Family engagement matters. For many CALD girls, families are not barriers but cultural anchors shaping norms around safety, modesty and acceptable forms of participation [5, 12]. Community organisations, faith‐based settings and women‐led cultural networks can strengthen trust and continuity.

Workforce capability also matters. Female role models from CALD backgrounds improve visibility and belonging, but representation alone is insufficient without teachers, coaches and practitioners trained in culturally responsive and non‐deficit approaches to engagement [12, 13].

Most importantly, future surveillance must move beyond asking whether CALD girls participate in sport and begin measuring how they move, how they load and what movement opportunities are genuinely available to them [6, 7, 11].

## 2 Conclusion

Physical inactivity among CALD girls in Australia is often discussed as a participation gap. Increasingly, it should also be understood as a surveillance gap. Current physical activity monitoring systems rely too heavily on self‐report, sport‐centric participation models and generic MVPA thresholds, which may underestimate meaningful movement exposure and fail to capture genuine developmental risk. This is particularly problematic during adolescence, where movement competence, confidence and mechanical loading exposure shape lifelong physical activity trajectories. If we continue to measure only what is easy to count, we will continue to miss what matters most.

CALD girls are not simply underrepresented in physical activity systems—they are often under‐measured within them. A prevention‐focused approach that combines objective monitoring, culturally grounded movement pathways, school‐based intervention, systems‐level accountability and methodological precision offers a stronger pathway forward. We cannot solve inequity if we continue measuring it inaccurately. A practical next step would be a formal review of the cultural validity of physical activity surveillance instruments currently used within Australian population monitoring systems. Organisations such as the AIHW, ABS and Sport Australia could play a central role in evaluating whether existing measures adequately capture the movement experiences of diverse populations, including CALD girls. Improving surveillance accuracy represents a critical foundation for more equitable policy, funding and intervention decisions.

## Author Contributions

The author conceptualised the commentary, conducted the literature review and wrote and revised the manuscript.

## Funding

The author has nothing to report.

## Conflicts of Interest

The author declares no conflicts of interest.

## Data Availability

Data sharing not applicable to this article as no datasets were generated or analysed during the current study.
